# A low resting heart rate at diagnosis predicts favourable long-term outcome in pulmonary arterial and chronic thromboembolic pulmonary hypertension. A prospective observational study

**DOI:** 10.1186/1465-9921-13-76

**Published:** 2012-09-03

**Authors:** Florian F Hildenbrand, Ivan Fauchère, Lars C Huber, Stephan Keusch, Rudolf Speich, Silvia Ulrich

**Affiliations:** 1 Department of Heart, Vessel, Thorax, University Hospital of Zurich, Zurich, 8091, Switzerland; 2 Department of Internal Medicine and Oncology, University Hospital of Zurich, Zurich, Switzerland; 3 Center for Integrative Human Physiology, University of Zurich, Zurich, Switzerland

**Keywords:** Chronic thromboembolic pulmonary hypertension, Heart rate, Prognosis, Pulmonary hypertension, Pulmonary arterial hypertension

## Abstract

**Background:**

A low resting heart rate (HR) is prognostically favourable in healthy individuals and in patients with left heart disease. In this study we investigated the impact of HR at diagnosis on long-term outcome in patients with differently classified precapillary pulmonary hypertension (pPH).

**Methods:**

pPH patients diagnosed as pulmonary arterial (PAH) or inoperable chronic thromboembolic pulmonary hypertension (CTEPH) were registered and regularly followed at our centre Baseline characteristics and events defined as either death or lung transplantation were noted. The prognostic value of HR was analysed using Kaplan Meier estimates, live tables and Cox regression.

**Results:**

206 patients with PAH (148) and inoperable CTEPH (58) were included. The median HR was 82 bpm. pPH with a HR below 82 bpm had a significantly longer overall event-free survival (2409 vs.1332 days, p = .000). This advantage was similarly found if PAH and CTEPH were analysed separately. Although a lower HR was associated with a better hemodynamic and functional class, HR was a strong and independent prognostic marker for transplant free survival even if corrected for age, sex, hemodynamics and functional status.

**Conclusion:**

We show that resting HR at diagnosis is a strong and independent long-term prognostic marker in PAH and CTEPH. Whether reducing HR by pharmacological agents would improve outcome in pPH has to be assessed by future trials with high attention to safety.

## Background

A lower resting heart rate (HR) is prognostically favourable in healthy individuals or in patients with cardiovascular disease [1-3]. An increased HR is an independent risk factor for cardiovascular and all-cause mortality in cardiovascular disease and left heart failure (LHF) [2,4]. In patients with systolic heart failure, the resting HR-threshold over which an increased mortality was found, was as low as 70 bpm [1].

The prognostic role of the HR in right sided heart failure due to precapillary pulmonary hypertension (pPH) is less clear. Studies have shown that an increased resting HR was a marker for disease burden and herewith associated with an unfavourable prognosis in patients with pulmonary arterial hypertension (PAH) [5,6]. Compared to healthy and to patients with left heart disease the cut off value for an adverse prognosis was higher in PAH (HR 82 bpm or 87 bpm in untreated and 92 bpm in specifically treated patients) [5,6]. The pathogenesis underlying a relatively increased HR in PAH is not completely understood. It is thought that the increased right ventricular afterload leads to an impaired right ventricular stroke volume. This reduced stroke volume will lead mainly via sympathetic activation to an increased HR, so that the cardiac output can be maintained in order to provide sufficient oxygen to the body, especially during exercise [7]. The increased HR in PAH might therefore rather be a consequence of disease burden and not an independent marker for adverse prognosis [6]. However, the increased sympathetic response along with the increased HR may unfavourably affect ventricular remodelling [5]. Until now it is not clear whether a lower HR is an independent prognostic factor in a broader pPH collective. We therefore aimed to investigate the prognostic value of the resting HR on long-term outcome in patients with differently classified pPH including PAH and inoperable chronic thromboembolic PH (CTEPH) [8,9].

## Methods

Patients who were diagnosed with pPH by right heart catheterisation (RHC) and classified in PAH or CTEPH by WHO [8] gave their written informed consent to have their data prospectively registered. Quality of life, NYHA/WHO functional class and 6 minute walk distance (6MWD) were assessed and registered at baseline [10]. During the diagnostic RHC, the following parameters were recorded as average of two separate measures during a stable period for at least 15 minutes recumbent rest: arterial blood pressures, resting HR, pulmonary artery pressures, right atrial pressure, pulmonary capillary occlusion pressure and cardiac output by continuous thermodilution. Mean pulmonary and systemic arterial pressures (mPAP and mAP), systemic and pulmonary vascular resistance (SVR and PVR) and cardiac index (CI) were calculated. Mixed venous and arterial blood samples were drawn for measures of oxygen saturations and partial pressures of oxygen and carbon dioxide. Patients were thereafter followed at our centre in close collaboration of specialists and general practitioners. Deaths of any cause and lung transplantation were defined as events. In June 2011 records of every single patient were reviewed for correct event recording and double-checked with the clinical report. If there were any doubt about events the general practitioner was contacted to assure life or occurrence of events. Patients with operable CTEPH who underwent pulmonary embolectomy were excluded from the survival analysis as the indication to operate is not merely correlated to disease severity.

The study was performed according to the Declaration of Helsinki, ICH-GCP as well as all national legal and regulatory requirements. National and international guidelines did not require institutional review board approval because this study was retrieved from our prospective registry.

### Statistics

All baseline data is summarized by mean ± standard deviation (SD) or medians (quartiles). Baseline variables by groups were compared using Mann-Witney-U-Test and Fisher-Test. Differential survival was assessed by Kaplan Meyer analysis and live tables, significance was tested by log-rank test and fishers test. Cox regression analysis was used for multivariate survival analysis. A p < 0.05 was considered significant. SPSS 19.0.0 (SPPS, Inc., Chicago Illinois) and Excel were used.

## Results

### Patients` characteristics

222 patients with pPH having had baseline evaluation with diagnostic RHC between January 1998 and June 2011 were included. 16 patients with subsequent pulmonary endarterectomy were excluded according to the protocol.

Characteristics of the remaining 206 pPH patients (130 females, age 55y ±17y) are shown in Table 1. 148 had PAH (55 idiopathic, 32 collagen vascular, 61 other associated) and 58 had inoperable CTEPH. Patients were in NYHA II to IV with a markedly reduced baseline 6MWD (386 ±137 m). The mean baseline resting HR was 82 ±14 bpm, the mPAP was 45 ±18 mmHg and PVR 775 ±464 dyn*s*m^-5^. Conventional medical treatment at baseline consisted of diuretics and oral anticoagulation. After baseline evaluation HR assessment, the following treatments had been started: Endothelin-1 receptor antagonists (85), Phosphodiesterase inhibitors (34), inhaled (63) or parenteral Prostanoids (8) and calcium antagonists (37, positive responders to acute vasodilator therapy, mostly switched to other therapies during follow-up.

**Table 1:** Patients’ baseline characteristics

	**Numbers (%) Mean ± SD**
Total number of patients	206
Female/male	130/76 (63/37)
Age (years)	55 ± 17
Precapillary Pulmonary arterial hypertension:	
-*Idiopathic*	55 (27)
-*Associated to collagen vascular disease*	32 (16)
-*Other associated PAH*	61 (30)
-*Chronic thromboembolic PH not eligible for surgery*	58 (28)
NYHA functional class I/II/III/IV	1/37/104/62 (1/18/51/30)^*^
BMI (kg/m^2^)	25 ±5
Mean resting heart rate (bpm)	82 ±14
Mean arterial pressure (mmHg) ^†^	90 ±14
Mean pulmonary arterial pressure (mmHg) ^†^	45 ±18
Pulmonary vascular resistance (dyn*sec*m^-5^) ^†^	775 ±464
Cardiac index (l/min/m^2^) ^†^	2.4 ±0.8
Right atrial pressure (mmHg) ^†^	9 ±6
NT-pro-BNP (ng/l, < 130)	1826 ±2269
6 minute walking distance (m)	386 ±137
Mean follow up (months)	45 ±35
Percentage survivors at year 1, 3, 5, 7, 9	84/67/57/55/53
Medication after baseline:	
-*Calcium antagonist*	37 (18)
-*Prostacyclin analogues (by inhalation)*	63 (31)
-*Prostacyclin analogues (parenteral)*	8 (4)
-*Endothelin-1 receptor antagonist*	96 (47)
-*Phosphodiesterase inhibitor*	34 (17)

* For two patients data were not registered,^*†*^*measured by right heart catheter;*

Data is given as number (%) or mean ± SD. NYHA: New York Heart Association, WHO: World health organization, BMI: Body mass index, NT-pro-BNP: NT-pro brain natriuretic peptide.

During the median follow-up of 37 (18;64) months, 24 patients died (18 PAH and 6 inoperable CTEPH), and 3 received lung transplantation (1 PAH and 2 CTEPH). Median overall survival was 4.8 years.

### ***Differences between pulmonary arterial and inoperable chronic thromboembolic pPH***

Patients with PAH were significant younger at baseline compared to CTEPH (51 ±18 vs. 61 ±12 y, p = 0.008) and had a higher mixed venous oxygen saturation (SmvO_2_ 61 ±11 vs. 57 ±10, p = 0.014). We found no other baseline differences and the overall event-free survival were similar in both classes (median 1776 vs. 1653 days, p = 0.47).

### Baseline and outcome difference according to resting heart rate

Patients characteristic for patients with resting HR below and above the median of 82 bpm are shown in Table 2. Patients with higher HR had less favourable hemodynamics, i.e. higher mPAP and PVR and lower SmvO2.

**Table 2:** Difference in baseline characteristics by median resting heart rate

	**< 82 bpm Numbers (%) Mean ± SD**	**> 82 bpm Numbers (%) Mean ± SD**	**p**
Total number of patients	101	105	0.780
Female/male	60/41 (59/41)	70/35 (67/33)	0.64/0.5
Age (years)	57 ±17	54 ±16	0.158
Idiopathic	24 (24)	31 (30)	0.345
Connective tissue disease	15 (15)	17 (16)	0.724
Other PAH	29 (29)	32 (31)	0.701
Chronic thromboembolic PH not eligible for surgery	33 (33)	25 (24)	0.294
WHO functional class I/II/III/IV	0/20/54/25 (20/54/25)	1/17/50/37 (16/48/35)	0.59/0.71/0.30
BMI (kg/m2)	25 ±5	25 ±6	0.545
Resting Heart Rate (bpm)	71 ±7	93 ±9	<0.001**
Mean arterial pressure (mmHg)^†^	88 ±14	91 ±14	0.116
Mean pulmonary arterial pressure (mmHg) ^†^	42 ±16	48 ±19	0.04*
Pulmonary vascular resistance (dyn*sec*m-5) ^†^	661 ±396	884 ±499	0.01*
Cardiac index (l/min/m2) ^†^	2.5 ±0.8	2.2 ±0.8	0.18
Right atrial pressure (mmHg) ^†^	7 ±5	11 ±7	<0.001**
NT-pro-BNP (ng/l, < 130)	1593 ±2079	2043 ±2429	0.283
6 minute walking distance (m)	409 ±141	366 ±131	0.11
Mean follow up (days)	1530	1179	0.15
Percentage survivors at year 1, 3, 5, 7, 9	93/78/68/64/62	77/57/49/48/47	0.40/0.18/0.19/0.23/0.28
Median survival (years)	6.6	3.7	<0.001**

* Significant,** highly significant^*†*^*measured by right heart catheter;*

Data is given as number (%) or mean ± SD. NYHA: New York Heart Association, WHO: World health organization, BMI: Body mass index, NT-pro-BNP: NT-pro brain natriuretic peptide.

**Figure 1:**
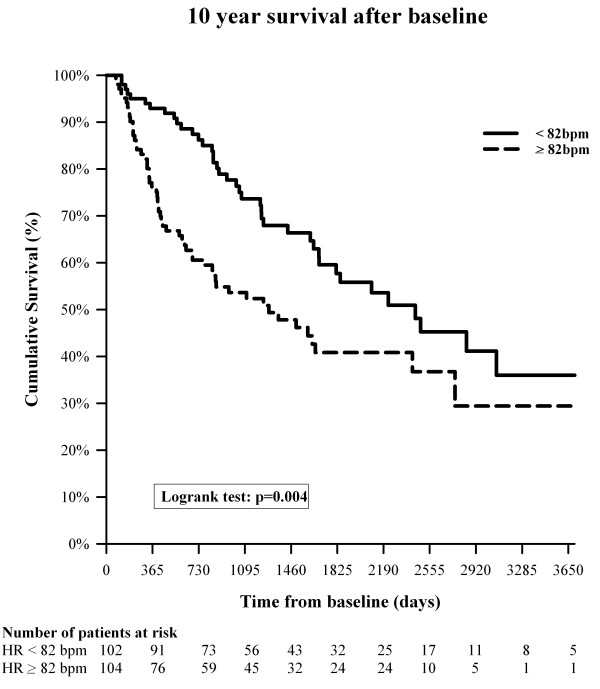
**10 year transplant free survival is shown for 206 patients with precapillary pulmonary hypertension.** Red line: patients with a baseline resting heart rate below 82 bpm. Blue line: patients with a baseline resting heart rate above 82 bpm, p = 0.004.

We found a significantly longer event-free survival for patients with a HR below 82 bpm (log rank p = 0.006, Figure 1). After 10 years, 61 (60%) patients with a baseline HR below 82 bpm were still alive compared with 48 (46 %) of patients with a HR ≥82 bpm. Consequently, the median transplant-free survival was significantly lower if the baseline HR was ≥82 bpm compared to <82 bpm (1581 vs. 2660 days, p < 0.0001). This significant event-free survival difference persisted even if PAH and CTEPH were analysed separately (p = 0.027 and p < 0.001). Analysis of the idiopathic and collagen vascular disease associated PAH subgroups revealed that a significant survival difference was found up to 8 and 5 years, respectively, but not thereafter due to the small number of cases at risk.

**Figure 2:**
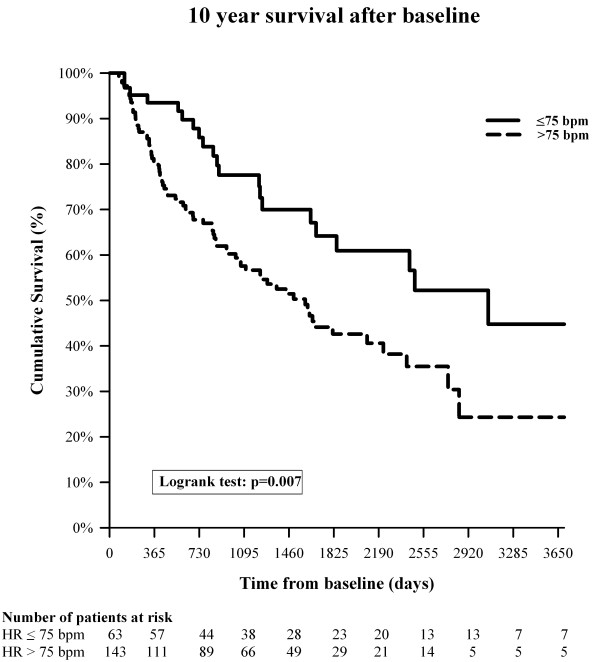
**10 year transplant free survival is shown for 206 patients with precapillary pulmonary hypertension.** Red line: patients with a baseline resting heart rate below 75 bpm. Blue line: patients with a baseline resting heart rate above 75 bpm, p = 0.007.

If, in analogy to left heart failure studies [1,3], lower cut off levels for the resting HR were taken, patients with lower resting HR cut offs had more favourable hemodynamics compared to patients above the cut off, Figure 2. Thus, for thresholds of 70 or 75 bpm, the mPAP measures were 40 ±14 and 42 ±13 compared with 49 ±17 and 50 ±17 mmHg (p = 0.002 and .008, respectively); the calculated PVR was 594 ±402 and 643 ±407 vs. 816 ±453 and 834 ±459 dyn*s*m^-5^ (p = 0.004 and p = 0.003). Median survival were 2008 and 1766 vs. 4028 and 3170 days for cut offs of 70 and 75 bpm (p = 0.087 and p = 0.002), respectively. The cumulative Kaplan Meyer survival differences did not reach statistical significance for the cut off of 70 bpm (log rank p = 0.069) but highly significant for the cut off of 75 bpm. A relatively high HR cut off of 92 bpm still revealed an advantage for patients with a HR below this value (p = 0.001).

### Multivariate survival analysis

In a multivariate Cox regression model including sex, age, PVR and HR, the resting HR was the strongest independent survival predictor (p < 0.0001). Baseline resting HR remained the strongest independent predictor of survival even when WHO functional class or the 6MWD were additionally included in the model (p < 0.0001 and p = 0.002). Resting HR persisted as strongest independent predictor even if the PVR was replaced by the cardiac index (p < 0.0001) or the right atrial pressure (p = .002) in the multivariate regression model.

## Discussion

In this long-time follow up collective of well characterized patients with PAH and CTEPH we found that a low resting HR at diagnosis was a strong and independent predictor of a favourable long-term outcome overall and for both groups separately. pPH patients with a resting HR below thresholds of 75 to 82 bpm had a markedly and significantly prolonged median survival compared to patients with higher resting HR. A lower resting HR remained an independent predictor of favourable outcome even if corrected for demographics, pulmonary hemodynamic, exercise capacity and functional class.

Many epidemiological studies in left heart failure have shown that a reduction of the HR improves cardiovascular outcome [1,3], implicating that an increased HR might not only be a mere compensatory mechanism, but, rather more, might represent an adverse factor on cardiac hemodynamics itself. The underlying mechanisms are not fully elucidated.

One explanation might be that the decreased diastolic filling time due to a shortened heart cycle does not allow sufficient myocardial perfusion to supply adequate oxygen to the diseased heart. Another reason might be that the augmented sympathetic activity leading to an increased HR is overshooting and, in turn, has adverse effects itself. The fact that not only sympathetic blockade by beta receptor blockers, but also mere HR reduction by Ion channel modification, result in a better outcome in patients with left heart failure points against the hypothesis of a single sympathetic adverse effect responsible for higher HR along with unfavourable outcomes [11,12].

In this study we could demonstrate that a lower resting HR is not only a favourable prognostic factor in patients with left heart failure, but also in patients with right heart failure due to differently classified pPH. Although patients with a lower resting HR had a better hemodynamic profile, functional class and relatively preserved exercise capacity, the fact that the resting HR remained an independent prognostic factor even if corrected for age, sex, pulmonary vascular resistance, exercise and functional capacities underscores that resting HR not only reflects the degree of disease burden but represents an independent predictor by itself in patients with pPH.

Although several studies linked a lower resting HR to longevity in healthy individuals and especially in patients with left heart disease [1,2,13,14], the assessment of HR thresholds, at which risk may increase, has not been the focus of studies until recent publications [1,15]. Moreover, the prognostic value of HR in major groups such as heart failure with preserved ejection fraction was only recently addressed [15]. These studies in LHF showed that a resting HR threshold as low as 70 bpm was prognostically discriminative. In the present study including patients with pPH, we tested several resting HR cut off points based on the median HR and in comparison with previously published cohorts of patients with left heart failure, where a cut off of 70 bpm was found [1], and a published cohort of idiopathic PAH, were a HR thresholds of 80 bpm was assessed [6]. In analogy to the later cohort, we found that a baseline resting HR above 82 bpm, the median in our cohort, was independently associated with increased mortality. We can therefore extend these published results to a broader and clinical relevant and realistic pPH population including associated PAH and CTEPH. Reducing the HR threshold to 75 bpm even better identified patients with a more favourable prognosis. A further threshold decrease to 70 bpm in analogy to published data in left heart disease, did not significantly differentiate the prognosis, most probably due to the small sample size, since only few patients in our cohort had resting HR below 70 bpm.

Recent guidelines do not include HR as prognostic marker, as the prognostic significance of HR in right heart failure due to pulmonary vascular disease had not been sufficiently studied [9,16]. Our data can fill this cap by showing that resting HR is an independent prognostic marker irrespective of demographics, hemodynamics and functional class. We herewith advocate that HR should be integrated in the clinical management, guidelines and trials including pPH patients.

At physiological pressures, the thin-walled, highly compliant RV can accommodate with high volumes (preload-dependency) [17]. However, in pPH the markedly increased RV afterload stresses the RV and results in inefficient work, especially during exercise. The capability to increase HR during exercise is therefore thought to be a key factor to maintain cardiac output. Therefore, most experts agreed for decades that agents which reduce HR and remove the body’s possibility to react with a chronotropic response towards the increased RV afterload are contraindicated in pPH [18,19]. In addition, the most frequently used agents to control HR, the beta adrenergic receptor blocker, could induce pulmonary vasoconstriction. However, several recent studies indicate that the beta-adrenergic system is over stimulated in pPH and this overstimulation may lead to a maladaptive RV remodelling as found in pPH [18,19]. Furthermore, supraventricular tachyarrhythmias are frequently found in pPH and associated with increased morbidity [20]. Beta adrenergic receptor blocker therapy have been shown to reduce mortality by about a third in left heart disease regardless of disease severity [21,22]. Reducing HR in left heart disease by this therapy reverses the maladaptive cardiac remodelling (at least partially), improves cardiac function and prevents arrhythmias [23]. However, these agents are either not mentioned in pPH guidelines or their use discouraged for fear of detrimental effects on hemodynamics due to the reduced chronotropic response, especially during exercise [16,24,25]. It may well be that these negative short-term effects of beta adrenergic blockage could be overcome by a reversal of the maladaptive cardiac remodelling, as suggested by data from a pPH animal model [19]. Theoretically, a HR decrease could also improve RV function by prolonging the relative duration of diastolic filling. This would allow a better coronary perfusion and oxygen delivery to the hypertrophied and often hypoperfused myocardium and may lead to an improved RV systolic function. Whether a treatment strategy aimed at slowing resting HR and reducing the increased beta-adrenergic response in pPH would be beneficial in pPH is still unclear. The results of our long-term study in pPH and other studies in idiopathic PAH showed that a reduced HR is prognostically important. Whether the medicamentous reduction of HR in pPH would be beneficial in pPH remains to be elucidated by future, well designed trial with special attention to the safety of such agents.

Our study is relatively small, however, pPH is a rare disease and we can provide robust long-term flow-up data in a thoroughly investigated and well followed pPH collective. Resting HR was carefully obtain after at least 10 minutes of rest with stable continuous hemodynamic curves on the monitor over at least 10 minutes. 

## Conclusions

In summary, our data show for the first time that baseline resting heart rate is a strong and independent long-term prognostic factor in a broad pPH collective including PAH and CTEPH. Whether reducing HR by pharmacological agents would improve outcome in pPH has to be assessed by future designed trials with high attention to safety.

## Abbreviations

6MWD: 6 minute walk distance; CI: Cardiac Index; CTEPH: Chronic Thromboembolic Pulmonary Hypertension; HR: Heart Rate; LHF: Left Heart Failure; mAP: Mean Arterial Pressure; mPAP: Mean Pulmonary Arterial Pressure; NYHA: New York Heart Association; PAH: Pulmonary Arterial Hypertension; pPH: Precapillary Pulmonary Hypertension; PVR: Pulmonary Vascular Resistance; RHC: Right Heart Catheterisation; SVR: Systemic Vascular Resistance; WHO: World Health Organisation.

## Competing interests

The authors declare that they have no competing interests.

## Authors' contributions

All authors had full access to the data and read and approved the final manuscript. *FFH* was involved in acquisition of data, analysis and interpretation of data, drafting the manuscript and revising it critically for important intellectual content and provided final approval of the version to be published. *IF* was involved in the interpretation of data and critical reading and revision of the draft manuscript, and final approval of the manuscript. *LCH* and *SK* made substantial contributions to the current study and publication, participating in the study process, analysis of data, development and revisions of the manuscript and has approved the final manuscript draft. *RS* was involved in the interpretation of data and critical reading and revision of the draft manuscript. *SU* developed the concept of this study, was involved in analysing the data, carried out the statistical analysis and participated in the interpretation, the critical revision of the study and final approval of the manuscript.
